# Prevalence and associated factors for stunting, underweight and wasting among children under 6 years of age in rural Hunan Province, China: a community-based cross-sectional study

**DOI:** 10.1186/s12889-022-12875-w

**Published:** 2022-03-11

**Authors:** Huixia Li, Shan Yuan, Hualing Fang, Guangwen Huang, Qun Huang, Hua Wang, Aihua Wang

**Affiliations:** 1 Department of Child Health Care, Hunan Provincial Maternal and Child Health Care Hospital, No.53, Xiangchun Road, Kaifu District, Changsha, 410008 China; 2 NHC Key Laboratory of Birth Defect for Research and Prevention, Hunan Provincial Maternal and Child Health Care Hospital, Changsha, Hunan Province China; 3 Department of Maternal Health Care, Hunan Provincial Maternal and Child Health Care Hospital, No.53, Xiangchun Road, Kaifu District, Changsha, 410008 China; 4 Department of Information Management, Hunan Provincial Maternal and Child Health Care Hospital, Changsha, Hunan Province China

**Keywords:** Children, Stunting, Underweight, Wasting, Prevalence, Rural, China

## Abstract

**Background:**

The existing epidemiological data cannot represent the situation of undernutrition among Chinese children, particularly those in rural China. Hence, in this community-based cross-sectional study, the prevalence and associated factors of stunting, underweight and wasting among children (age < 6 years) from rural Hunan Province were analyzed.

**Methods:**

Totally 5529 children aged 0 to 71 months and their caregivers were randomly chosen by multistage stratified cluster sampling from 72 villages from rural Hunan, which were distributed in 24 towns of 12 counties. Data about the children and their mothers, caregivers and family conditions was acquired using unified questionnaire, and the length/height and weight of each child were measured using unified instruments. The prevalence of undernutrition among children was evaluated using the length/height for age, weight for age, weight for length/height, and body mass index for age z scores, which were computed according to the 2006 and 2007 WHO Child Growth Standards.

**Results:**

The prevalence of stunting, underweight, and wasting among the 5529 children were 4.4% (241), 3.9% (217), and 4.0% (221), respectively. The significant associated factors on higher risks of undernutrition in the children were low birth weight, maternal gestational weight gain <10 kg (stunting); low birth weight, maternal gestational weight gain <10 kg, ethnicity of caregivers being minority, large family size (underweight); low birth weight, ethnicity of caregivers being minority, large family size (wasting). High education level of caregivers and high family food expenditure were common protective factors for all three types of undernutrition, except that high family food expenditure was not protective against wasting.

**Conclusions:**

The prevalence of stunting, underweight and wasting is low among rural children under age of 6 years in Hunan. As for the measures, the gestational care and reasonable diet of mothers should be strengthened, and nutritional deficiency during pregnancy be avoided, which will prevent low birth weight. The local economic development and the education level of caregivers need to be further improved, especially for minorities.

## Background

The malnutrition of children includes undernutrition, over-nutrition and micronutrient deficiency. Of them, undernutrition consists of three indices of stunting, underweight and wasting. In particular, stunting and wasting reflect the chronic or acute undernutrition of children, and underweight reveals the current undernutrition status, but cannot differentiate near-term or long-term undernutrition [1]. Different forms undernutrition may coexist in children. Preschool children are at a critical period of growth and development, and undernutrition occurring at this period may cause irreversible near-term and long-term effects on the health of children. Such effects include delayed physical and cognitional development [2–4], which even increase the risks of infection, death, hypertension, diabetes and other chronic diseases at adulthood [5].

About 149 million children under 5 years of age suffered stunting and about 49 million children had wasting in 2018 according to data from United Nations Children's Fund (UNICEF) and World Health Organization (WHO) [6]. Undernutrition of children is a major public health problem in developing countries, especially Africa and Southeast Asia. China is a developing country in East Asia. With the rapid socioeconomic development in the past 30 years, the significant increase in average disposable income, the optimization of dietary structure, the improvement of caregivers education level and the medical and health services, the nutrition status of children has been significantly improved [5, 7]. Nationwide nutrition surveys in China show that the prevalence of stunting and underweight among children under 5 years of age between 1990 and 2010 dropped from 33.1% to 9.9% and from 13.7% to 3.6% respectively, but were still up to 20.3% and 8.0% respectively in rural China [5, 7].

In China, the vast majority of poor areas are concentrated in the rural areas of the central and western provinces. Hunan province with medium economic development is located in central China, and the administrative region includes 123 districts/counties in 14 cities. Hunan is dominated by rural population, accounting for 65.1% [8]. Hunan is a multiracial province and is dwelt by 9.5% of minorities, including Miao, Tujia, and Dong. In rural Hunan, the economic development is relatively poor, and the caregivers have low education level and deficient nutritional knowledge, and many children receive unscientific feeding and suffer from undernutrition [9, 10]. A cross-sectional study conducted in 2009 showed that the prevalence of stunting and underweight among children aged 0-7 years in rural Hunan were 16.6% and 7.8%, respectively [11], which were obviously higher than the national average in 2010. Undernutrition of children can be caused by many factors. A number of observational studies show that poor socioeconomic development, inappropriate complementary feeding practices, household food insecurity, diseases of children (e.g. recurrent respiratory infections and diarrhea), inadequate maternal nutritional status, low education level of caregivers, and children left behind by internal migration are all associated with the undernutrition of children [12–17].

To further improve the nutrition status of children in poor rural areas, National Health Commission of China extensively implemented children nutrition improvement projects in poor rural areas including Hunan province since 2012, including food supplements and nutrition education [18]. So far, children nutrition improvement projects have been implemented in rural areas of Hunan for eight years. Hence, previous epidemiological investigations are already unable to reflect the current undernutrition levels of children in rural Hunan. Though several children undernutrition investigations have been conducted in rural Hunan recently, the investigated age ranges are narrow and concentrated on under age of 3 years [19–21]. In particular, relevant research on preschool children is insufficient [22].

In the new era, systematic study on the undernutrition of children under 6 years of age in rural areas, and discovery of key associated factors are critical for prevention and treatment of undernutrition among children in rural areas. Hence, in this study, a community-based cross-sectional survey was conducted to clarify the status and associated factors of undernutrition among children under 6 years of age in rural Hunan. Some targeted interventions were proposed to improve the nutrition status of rural children.

## Materials and methods

### Subjects

The subjects were children under 6 years of age (0 to 71 months) and their caregivers from rural Hunan investigated between August and November 2019. The sample size was determined according to relevant equations for cross-sectional studies [23]. Since the stunting prevalence among rural children was estimated to be 16 %[11], the size of a test α was 0.05, permissible error *d* was 0.10, the designed effect of complex samples was 2 and the non-response rate was 20%, the final sample size was determined to be 5040 (= 2100×2× 1.2).

Subjects were selected by multistage stratified cluster sampling. In China, one province consists of several cities, and one city consists of several districts and several counties (districts and counties are urban and rural, respectively); one county contains several towns, and one town has several villages [22]. The 14 cities in Hunan were divided by economic development into three levels: high, moderate and low. Then 2 cities from each economic level, 2 counties from each selected city, 2 towns from each selected county, and 3 villages from each selected town were randomly selected. From each village, all eligible children were included into our subjects. Totally, 5529 children from 72 villages covering 24 towns in 12 counties were involved.

### Data collection

This study consisted of a questionnaire survey and anthropometric measurements. The questionnaire included children’s factors (gender, age, birth weight, preterm birth, left-behind children, only child, passive smoking, regular physical examination), maternal gestational factors (age at delivery, gestational gain weight, moderate/severe anemia, pregnancy comorbidity), and factors of caregivers and family (type of caregivers, ethnicity, education level, occupation, family size, family income, family food expenditure). All children received anthropometric measurements, including length/height, and weight.

#### Definition of variables

Birth weight referred to the infant’s net weight within 1 hour of birth, and did not include any wear or wraps, which can be obtained from the child’s vaccination certificate or medical certificate of birth. If there was no vaccination certificate or medical certificate of birth, the birth weight of the child was obtained from the recalling by the caregivers. The birth weight <2500 g, 2500-3999 g, and ≥ 4000 g were considered as low birth weight, normal birth weight, and macrosomia, respectively. Birth at <37 gestational weeks was regarded as premature birth. Left-behind children referred to those children whose parents (both or either) worked in other places and did not live together with the children [22]. Only child was the only child born by one couple, and had no siblings. Passive smoking meant a nonsmoker inhaled at least 15 minutes every day the smoke exhaled by smokers for at least 1 day within 1 week. Maternal gestational weight gain was determined by the final weight of the mother measured in late pregnancy before delivery subtracted by the weight in early pregnanc y[24] and was divided into four groups of <10.00, 10.00-14.99, 15.00-19.99, and ≥20.00 kg. Maternal moderate/severe anemia was defined as a hemoglobin level <100 g/ L[25]. The maternal hemoglobin concentration in this study was the concentration in the third trimester of pregnancy. The maternal weights in early pregnancy and in late pregnancy before delivery, and the hemoglobin concentration in the third trimester of pregnancy can be obtained from the clinical medical records of maternal health handbook. If there was no maternal health handbook, the maternal weights and hemoglobin concentration during pregnancy were obtained from the recalling by the caregivers. The maternal pregnancy comorbidities included gestational diabetes mellitus, gestational hypertension, pregnancy associated with cardiac diseases, gestational liver diseases, and thyroid dysfunction. Caregivers were those who took care of the diets, living and personal security of children and were divided into two types: parents, and grandparents/others. Ethnicity of caregivers was divided into Han and minorities. The education level of caregivers was classified into primary school or below, junior high school, senior high school, and college or above. The occupation of caregivers was divided into housework, government agencies staff, business service staff, farmer, and others. Family size was defined as the total number of family members and involved the members with economic relations and joint budget and diets, and was separated into ≤4, 5-6, and ≥7.

#### Anthropometric measurements

The investigators used unified instruments to measure the length/heights and weights of children according to standardized methods, which were described by the *Technical Specification for Children Health Check Service* (China Ministry of Health, 2012). The lengths and weights of children aged 0-23 months were measured by using an FSG-25-YE lying-form infants and young children precision medical examination meter (Shanghai Betterren Medical Tech Co., Ltd.). The heights and weights of children aged 24-71 months were measured using an HX-200 stadiometer and an HCS-50-RT electronic scale respectively (Liheng Instrumentation LTD., Shanghai, China). The accuracies of instruments for length/heights and weights were 0.1 cm and 0.05 kg, respectively.

#### Evaluation criteria for children physical development

The commonly-used indices for children physical development are length/height for age, weight for age, weight for length/height, and body mass index (BMI) for age. BMI was calculated using the ratio between a child's weight in kilograms and length/height in meters squared (kg/m^2^): BMI = weight (kg)/ height^2^ (m^2^). A child’s physical development was evaluated using Z-score recommended by WHO: Z score = (analyzed index - median of reference standard)/standard deviation of reference standard. The WHO Child Growth Standards involve two age groups: 0-5 years (0-60 months) and 5-19 years (61-228 months), which are 2006 Child Growth Standar d[26] and 2007 Child Growth Standar d[27]. Hence, the physical development of children was evaluated according to the two age groups above.

1. Children aged 0-60 months: length/height for age z score (HAZ), weight for age z score (WAZ) and weight for length/height z score (WHZ) were calculated according to WHO 2006 Child Growth Standard. HAZ <-2, WAZ <-2 and WHZ <-2 were defined as stunting, underweight, and wasting respectively.
2. Children aged 61-71 months: HAZ, WAZ, and BMI for age z score (BMIZ) were calculated according to WHO 2007 Child Growth Standard. HAZ <-2, WAZ <-2 and BMIZ <-2 were defined as stunting, underweight, and wasting respectively.

### Quality control

The investigators were the child health care doctors selected from the county-level maternal and child health care hospitals of the corresponding counties. The investigators conducted a face-to- face interview with children’s caregivers. Prior to the survey, all the investigators were trained unifiedly, and only the qualified ones were allowed to take part in on-site survey. The instruments were calibrated before and during investigations. The physique measuring staff measured the length/height and weight of children in strict accordance with the specifications of the instruments. During the survey, all copies of the questionnaire were checked by a quality controller. Each copy should be filled in in a complete and standard way. Any illogical or missed response should be corrected in time. Data were double-inputted on Epidata 3.1 and tested in terms of consistency. For any inconsistent data, the original copy should be checked to ensure the high quality of any inputted data.

## Statistical analysis

HAZ, WAZ, WHZ and BMIZ were computed using WHO anthropometric macros in SPSS (igrowup_SPSS and WHO2007_SPSS) [28, 29], and statistical analyses were conducted on SPSS 25.0 (IBM, Chicago, IL, USA). Categorical data was statistically described as proportion or rate. The prevalence of stunting, underweight and wasting among children with different characteristics was compared by Chi-square test. With undernutrition status (stunting, underweight, wasting) as dependent variables (No=0, Yes=1), all 19 factors related to the characteristics of children, mothers, caregivers and families were used as independent variables, and multivariate logistic regression models were developed to identify the associated factors of children’s undernutrition. Variables were selected using a backward selection method. Multivariate logistic regression models were developed separately for each undernutrition status: stunting, underweight, and wasting. The strength of association between significant variables and undernutrition was evaluated by using odds ratios (ORs) with 95% confidence interval (CI). All statistical tests were two-tailed, and the significant level was *P*<0.05*.*

### Ethics approval and consent to participate

The study protocol was approved by the Ethics Committee of Hunan Provincial Maternal and Child Health Care Hospital (No.2019-S036). The study was conducted in accordance with the Declaration of Helsinki. Written informed consents were obtained from all the caregivers of children involved in this study.

## Results

### Characteristics of children

Totally 5800 copies of the questionnaire were sent out, and 5645 copies were returned, with a reply rate of 97.3%. Of them, 5529 copies were valid, with a valid rate of 97.9%. Of the 5529 children investigated, 50.8% were boys and 49.2% were girls (Table 1). The major age groups were 48- 59 and 60-71 months old, which accounted for 20.8% and 20.1% respectively. The proportions of low birth weight, preterm birth, left-behind children, only child, passive smoking and regular physical examination were 3.7%, 4.8%, 42.4%, 24.7%, 43.8% and 88.5% respectively.

**Table 1:** Characteristics of children under 6 years old who participated in the study

Characteristics	Frequency (n)	Percentage (%)
Sex of children		
Female	2723	49.2
Male	2806	50.8
Age of children (months)		
0-11	716	12.9
12-23	800	14.5
24-35	835	15.1
36-47	914	16.5
48-59	1150	20.8
60-71	1114	20.1
Birth weight		
<2500 g	203	3.7
2500-3999 g	4968	89.9
≥4000 g	358	6.5
Preterm birth		
No	5264	95.2
Yes	265	4.8
Left behind children		
No	3184	57.6
Yes	2345	42.4
Only child		
No	4157	75.2
Yes	1372	24.8
Passive smoking		
No	3110	56.2
Yes	2419	43.8
Regular physical examination		
No	634	11.5
Yes	4895	88.5

### Gestational conditions of mothers

The maternal age at delivery was mainly 25-29 years and 30-34 years, accounting for 30.8% and 40.8% respectively (Table 2). The proportions of maternal gestational weight gain <10.00 kg, moderate/severe anemia, and pregnancy comorbidity were 19.1%, 4.9% and 9.9% respectively.

**Table 2:** Characteristics of children’s mothers during pregnancy

Characteristics	Frequency (n)	Percentage (%)
Maternal age at delivery (years)		
<20	25	0.5
20-24	394	7.1
25-29	1702	30.8
30-34	2258	40.8
35-39	806	14.6
≥40	344	6.2
Maternal gestational weight gain (kg)		
<10.00	1058	19.1
10.00-14.99	2356	42.6
15.00-19.99	1402	25.4
≥20.00	713	12.9
Maternal moderate/severe anemia		
No	5258	95.1
Yes	271	4.9
Pregnancy comorbidity		
No	4982	90.1
Yes	547	9.9

### Characteristics of caregivers and family

As shown in Table 3, the caregivers were mostly parents (67.1%). The ethnicity of caregivers was mostly Han (90.5%), the education level was mainly junior middle school (38.4%), and the dominant occupation was housework (57.4%). The family size was mostly 5-6 members (53.6%), the family annual income was mainly ≥60000 (61.6%), and the family annual food expenditure was mostly ≥6000 Yuan (72.9%).

**Table 3:** Characteristics of caregivers and family

Characteristics	Frequency (n)	Percentage (%)
Type of caregivers		
Parents	3711	67.1
Grandparents/other	1818	32.9
Ethnicity of caregivers		
Han	5005	90.5
Minorities	524	9.5
Education level of caregivers		
Primary school or below	1116	20.2
Junior high school	2121	38.4
Senior high school	1248	22.6
College or above	1044	18.9
Occupation of caregivers		
Housework	3175	57.4
Government agencies staff	673	12.2
Business services staff	457	8.3
Farmer	355	6.4
Other	869	15.7
Family size		
≤4	1644	29.7
5-6	2963	53.6
≥7	922	16.7
Family income ( Yuan/year)		
<20000	237	4.3
20000-39999	612	11.1
40000-59999	1275	23.1
≥60000	3405	61.6
Family food expenditure (Yuan/year)		
<2000	127	2.3
2000-3999	585	10.6
4000-5999	789	14.3
≥6000	4028	72.9

### Prevalence of stunting, underweight, and wasting among children

The prevalence of stunting, underweight, and wasting among the children were 4.4% (241/5529), 3.9% (217/5529), and 4.0% (221/5529) respectively. Table 4 shows a comparison of prevalence of stunting, underweight, and wasting in children with different characteristics. The prevalence of stunting significantly differed with birth weight, preterm birth, maternal gestational weight gain, ethnicity of caregivers, education level of caregivers, and family food expenditure groups (*P*<0.05). The prevalence of underweight significantly differed with birth weight, preterm birth, maternal gestational weight gain, ethnicity of caregivers, education level of caregivers, family income, and family food expenditure groups (*P*<0.05). The prevalence of wasting significantly differed with birth weight, preterm birth, regular physical examination, ethnicity of caregivers, education level of caregivers, family size, and family food expenditure (*P*<0.05).

**Table 4:** The prevalence of undernutrition according to children, mothers, caregivers and family characteristics

Characteristics	Frequency	Undernutrition, n (%)					
		Stunting	*P*^a^	Underweight	*P*^b^	Wasting	*P*^c^
**Children levels**							
Sex of children			0.053		0.341		0.983
Female	2723	104(3.8)		100(3.7)		109(4.0)	
Male	2806	137(4.9)		117(4.2)		112(4.0)	
Age of children (months)			0.236		0.599		0.057
0-11	716	22(3.1)		21(2.9)		17(2.4)	
12-23	800	43(5.4)		31(3.9)		24(3.0)	
24-35	835	36(4.3)		30(3.6)		33(4.0)	
36-47	914	39(4.3)		35(3.8)		39(4.3)	
48-59	1150	58(5.0)		52(4.5)		54(4.7)	
60-71	1114	43(3.9)		48(4.3)		54(4.8)	
Birth weight			<0.001^*^		<0.001^*^		<0.001^*^
<2500 g	203	27(13.3)		27(13.3)		21(10.3)	
2500-3999 g	4968	207(4.2)		187(3.8)		194(3.9)	
≥4000 g	358	7(2.0)		3(0.8)		6(1.7)	
Preterm birth			<0.001^*^		<0.001^*^		<0.001^*^
No	5264	216(4.1)		191(3.6)		199(3.8)	
Yes	265	25(9.4)		26(9.8)		22(8.3)	
Left behind children			0.768		0.480		0.553
No	3184	141(4.4)		130(4.1)		123(3.9)	
Yes	2345	100(4.3)		87(3.7)		98(4.2)	
Only child			0.562		0.437		0.542
No	4157	185(4.5)		168(4.0)		170(4.1)	
Yes	1372	56(4.1)		49(3.6)		51(3.7)	
Passive smoking			0.953		0.333		0.431
No	3110	136(4.4)		129(4.1)		130(4.2)	
Yes	2419	105(4.3)		88(3.6)		91(3.8)	
Regular physical examination			0.367		0.399		0.044^*^
No	634	32(5.0)		21(3.3)		16(2.5)	
Yes	4895	209(4.3)		196(4.0)		205(4.2)	
**Mothers levels**							
Maternal age at delivery (years)			0.803		0.069		0.119
<20	25	2(8.0)		3(12.0)		2(8.0)	
20-24	394	20(5.1)		20(5.1)		26(6.6)	
25-29	1702	79(4.6)		76(4.5)		67(3.9)	
30-34	2258	92(4.1)		70(3.1)		82(3.6)	
35-39	806	35(4.3)		32(4.0)		34(4.2)	
≥40	344	13(3.8)		16(4.7)		10(2.9)	
Maternal gestational weight gain (kg)			0.015^*^		0.040^*^		0.072
<10.00	1058	63(6.0)		52(4.9)		55(5.2)	
10.00-14.99	2356	95(4.0)		96(4.1)		89(3.8)	
15.00-19.99	1402	62(4.4)		53(3.8)		45(3.2)	
≥20.00	713	21(2.9)		16(2.2)		32(4.5)	
Maternal moderate/severe anemia			0.954		0.907		0.710
No	5258	229(4.4)		206(3.9)		209(4.0)	
Yes	271	12(4.4)		11(4.1)		12(4.4)	
Pregnancy comorbidity			0.684		0.205		0.114
No	4982	219(4.4)		201(4.0)		206(4.1)	
Yes	547	22(4.0)		16(2.9)		15(2.7)	
**Caregivers and family levels**							
Type of caregivers			0.220		0.327		0.355
Parents	3711	153(4.1)		139(3.7)		142(3.8)	
Grandparents/other	1818	88(4.8)		78(4.3)		79(4.3)	
Ethnicity of caregivers			0.003^*^		<0.001^*^		<0.001^*^
Han	5005	205(4.1)		177(3.5)		163(3.3)	
Minorities	524	36(6.9)		40(7.6)		58(11.1)	
Education level of caregivers			0.001^*^		0.003^*^		<0.001^*^
Primary school or below	1116	63(5.6)		54(4.8)		64(5.7)	
Junior high school	2121	106(5.0)		96(4.5)		93(4.4)	
Senior high school	1248	46(3.7)		45(3.6)		36(2.9)	
College or above	1044	26(2.5)		22(2.1)		28(2.7)	
Occupation of caregivers			0.105		0.795		0.138
Housework	3175	157(4.9)		129(4.1)		132(4.2)	
Government agencies staff	673	20(3.0)		23(3.4)		19(2.8)	
Business services staff	457	15(3.3)		16(3.5)		12(2.6)	
Farmer	355	16(4.5)		17(4.8)		15(4.2)	
Other	869	33(3.8)		32(3.7)		43(4.9)	
Family size			0.307		0.054		0.039^*^
≤4	1644	62(3.8)		51(3.1)		56(3.4)	
5-6	2963	133(4.5)		120(4.0)		115(3.9)	
≥7	922	46(5.0)		46(5.0)		50(5.4)	
Family income ( Yuan/year)			0.080		0.010^*^		0.094
<20000	237	14(5.9)		17(7.2)		15(6.3)	
20000-39999	612	35(5.7)		32(5.2)		27(4.4)	
40000-59999	1275	61(4.8)		50(3.9)		58(4.5)	
≥60000	3405	131(3.8)		118(3.5)		121(3.6)	
Family food expenditure (Yuan/year)			0.004^*^		0.008^*^		0.002^*^
<2000	127	10(7.9)		8(6.3)		5(3.9)	
2000-3999	585	39(6.7)		36(6.2)		40(6.8)	
4000-5999	789	34(4.3)		33(4.2)		35(4.4)	
≥6000	4028	158(3.9)		140(3.5)		141(3.5)	
Total	5529	241(4.4)		217(3.9)		221(4.0)	

^a^The comparison of stunting prevalence in children with different characteristics

^b^The comparison of underweight prevalence in children with different characteristics

^c^The comparison of wasting prevalence in children with different characteristics

^*^P<0.05

### Associated factors of stunting, underweight, and wasting among children

Multivariate logistic regression analyses showed that low birth weight (<2500 g) and maternal gestational weight gain <10.00 kg were significantly associated with an increased risk of stunting in the children (AOR=3.44, 95%CI: 2.23-5.31; AOR=1.74, 95%CI: 1.04-2.90). High education level of caregivers and high family food expenditure were protective factors against stunting in the children (AOR=0.80, 95%CI: 0.69-0.92; AOR=0.85, 95%CI: 0.73-0.99). Low birth weight (<2500 g), maternal gestational weight gain < 10.00 kg, ethnicity of caregivers being minority and large family size were significantly associated with an increased risk of underweight in the children (AOR=2.86, 95%CI: 1.67-4.90; AOR=1.83, 95%CI: 1.03-3.26; AOR=1.97, 95%CI: 1.35-2.86; AOR=1.26, 95%CI: 1.03-1.55). High education level of caregivers and high family food expenditure were protective factor against underweight in the children (AOR=0.80, 95%CI: 0.69-0.93; AOR=0.86, 95%CI: 0.73-0.98). Low birth weight (<2500 g), ethnicity of caregivers being minority and large family size were significantly associated with an increased risk of wasting in the children (AOR=2.22, 95%CI: 1.24-3.98; AOR=3.45, 95%CI: 2.50-4.77; AOR=1.23, 95%CI: 1.01-1.50). High education level of caregivers was a protective factor against wasting in the children (AOR=0.81, 95%CI: 0.70-0.93) (Table 5).

**Table 5:** Factors associated with children undernutrition in multivariate analysis

Factors	Stunting				Underweight				Wasting			
	*β*	*P*	AOR	95%CI	*β*	*P*	AOR	95%CI	*β*	*P*	AOR	95%CI
Birth weight												
<2500 g	1.24	<0.001^*^	3.44	2.23-5.31	1.05	<0.001^*^	2.86	1.67-4.90	0.80	0.007^*^	2.22	1.24-3.98
2500-3999 g			1.00				1.00				1.00	
≥4000 g	-0.73	0.060	0.48	0.22-1.03	-1.53	0.09^*^	0.22	0.07-0.68	-0.84	0.047^*^	0.43	0.19-0.99
Maternal gestational weight gain (kg)												
<10.00	0.55	0.035^*^	1.74	1.04-2.90	0.60	0.041^*^	1.83	1.03-3.26				
10.00-14.99	0.22	0.372	1.25	0.77-2.03	0.51	0.066	1.67	0.97-2.87				
15.00-19.99	0.39	0.134	1.48	0.89-2.45	0.49	0.091	1.64	0.92-2.91				
≥20.00			1.00				1.00					
Ethnicity of caregivers												
Han			1.00				1.00				1.00	
Minorities	0.35	0.074	1.42	0.97-2.07	0.68	<0.001^*^	1.97	1.35-2.86	1.24	<0.001^*^	3.45	2.50-4.77
Education level of caregivers	-0.23	0.001^*^	0.80	0.69-0.92	-0.22	0.004^*^	0.80	0.69-0.93	-0.22	0.003^*^	0.81	0.70 -0.93
Family size					0.23	0.026^*^	1.26	1.03-1.55	0.21	0.048*	1.23	1.01-1.50
Family food expenditure (Yuan/year)	-0.16	0.041^*^	0.85	0.73-0.99	-0.15	0.048^*^	0.86	0.73-0.98				
Constant	-2.77	<0.001^*^		<0.001^*^	-3.32	<0.001^*^	0.04		-4.54	<0.001^*^	0.01	

*AOR *adjusted odds ratio; *CI *confidence interval; ^*^P<0.05

The likelihood ratio test showed that multivariate logistic regression models for stunting, underweight, and wasting were statistically significant (χ^2^=68.766, *P*<0.001; χ^2^=83.485, *P*<0.001; χ^2^=96.773, *P*<0.001)

## Discussion

### Undernutrition status of children

This study shows that the prevalence of stunting, underweight and wasting among children under 6 years of age in rural Hunan are 4.4%, 3.9% and 4.0% respectively, which are lower compared with the majority of developing countries, especially Southeast Asia (e.g. Pakistan, Bhutan, Myanmar, India), and are close to those of developed countries, such as the US A[15, 30–33]. A national family health investigation in India during 2015-2016 showed that the prevalence of stunting and underweight among children under 5 years of age were up to 38% and 35% respectivel y[33]. The prevalence of stunting and underweight among children under 6 years of age in rural Hunan reached the requirements of the *China Children Development Outline (2011-2020)* (be lower than 7% and 5%, respectively).

The possible reason for the low undernutrition prevalence in this study is that the Chinese government has implemented child nutrition improvement projects in poor rural areas recently. As one of the few provinces to first implement the national child nutrition improvement project in poor rural areas of China, the project in 2012 covered 20 counties of Hunan, expanded to 25 counties in 2014, and adjusted to 53 counties in 2018, which covered all poor villages of Hunan. Specifically, all children aged 6-23 months in poor rural areas were provided for free with 1 bag/day of complementary food supplement (Yingyangbao, YYB for short) containing 6 vitamins and 3 minerals (Ca, Fe, Zn), and childhood nutrition knowledge was propagandized to the caregiver s[18].

### Associated factors of undernutrition in children

#### Birth weight

Birth weight is a key indicator about the intrauterine nutrition status of the fetus, and is one of the critical factors that decide postnatal growth and development. A cross-sectional study in Iran demonstrates that the physical development levels (e.g. length, weight, head circumference) of infants with low or extremely low birth weight are significantly lower at the 18-th month compared with infants with normal birth weight [34]. Several observational studies confirm that low birth weight is an independent risk factor of undernutrition in children [15, 35, 36]. The present study finds that low birth weight is the most important influence factor on the undernutrition of children in rural Hunan, and the risks of children with low birth weight to suffer stunting, underweight and wasting are 3.44, 2.86 and 2.22 times those of children with normal birth weight, respectively. Infants with low birth weight more commonly suffer preterm birth or intrauterine growth retardation, and the functions of their organs/systems are undeveloped, such as imperfect sucking and swallowing abilities, poor digestion and absorption, and difficulties in feeding. Moreover, these infants have low autoimmunity and are susceptible to the harmful external environment and diseases, which can lead to the delayed growth and development [37].

#### Maternal gestational weight gain

The gestational nutrition of the mother is closely related to the child's growth and development [15, 24, 38]. A retrospective cohort study in China shows that the pre-pregnant BMIs and gestational weight gains of mothers are both positively correlated with the WAZ and HAZ of the children aged 12 month s[38]. Data analysis of a National Demographic and Health Survey in Myanmar during 2015-2016 shows that maternal nutritional inadequacy will raise the risks of underweight and wasting among children under 5 years of ag e[15]. Similarly, our study demonstrates that maternal gestational weight gain is related to the undernutrition of children, and weight gain <10.00 kg will increase the risks of stunting and underweight in children by 74% and 83% respectively, but we report no association between maternal gestational weight gain and wasting of children. Though studies show that the increase of maternal gestational weight gain will reduce the risk of undernutrition in children [24], it does not mean the larger weight gain is better, which is because excessive gestational weight gain will in turn increase the risk of overweight/obesity in children [39, 40]. Hence, providing mothers with regular prenatal care and reasonable diet to avoid gestational excessive or insufficient weight gain is significant for malnutrition prevention in children.

#### Ethnicity of caregivers

Hunan is a multiracial province in Central China and is dwelt by 9.5% of minorities, including Miao, Tujia, and Dong. Though previous studies show the ethnicity of the mother or caregivers is related to the undernutrition of children, the findings are inconsistent [19, 22]. Zhou et al. show that the ethnicity of Miao or Tujia is a protective factor against undernutrition in children, as the prevalence of undernutrition in Miao and Tujia children is lower than that of Han children [19]. A cross-sectional study in rural poor areas of China suggests that ethnicity of Tibetan, Yi or other minorities is a risk factor of stunting in children, as the risks of stunting are 2.35, 1.95 and 1.42 times higher compared with Han childre n[22]. Our findings are different from the above two studies and indicate that ethnicity of minority is a risk factor of underweight and wasting in children, but is not related to stunting. These differences may be attributed to two reasons. Firstly, the economy, cultures, religions, living conditions and life customs are all different among ethnics. Secondly, the sample sizes, definitions of indices, and data analyses are all different among studies, as Zhou et al. concerned the total prevalence of undernutrition, but ignored the types of undernutrition. In our study, the education level of caregivers with minority ethnicity is significantly lower than that of Han caregivers, and the family income and family food expenditure of minority ethnicity are also significantly lower than those of Han. Caregivers with low education level often lack nutritional knowledge, and families under poor socio-economic status have low food consumption capacity, both of which will directly affect the quality of children’s diet, thereby increasing the risk of undernutrition in children.

#### Education levels and family incomes

Many observational studies confirm that high education level of mothers or caregivers and high family income are two protective factors against undernutrition of childre n[7, 32, 35, 36, 41, 42]. Our study shows that as the education level of caregivers and the family food expenditure increase, the risk of undernutrition in children is lowered, which is consistent with other studies. This is because the increased education level of caregivers can largely improve their acceptance of nutrition and health knowledge, which thereby promotes the scientific feeding and diet balancing of children [5]. Generally, family food expenditure will not increase unless the family incomes rise, and thus is the most direct and objective reflection of family incomes. Since family incomes and family food expenditure are collinear, the variable of family income was not involved in our multivariate logistic regression models. Nevertheless, the protective effect of family food expenditure on undernutrition among children directly reflects the protective effect of family incomes on undernutrition.

#### Family size

In addition, this study also finds that as the family size increases, the risk of underweight and wasting in children is increased. This finding is consistent with the study conducted in Ethiopi a[13] which shows that the risk of wasting is 1.22 times higher among children who have lived in family members of 6-10 than children who have lived in family members of 1-5. The increase in family size will increase the financial burden of the whole family, and therefore inevitably affect the nutritional status of children.

## Limitations

This cross-sectional study has some limitations. First, the relationship between investigated factors and undernutrition is statistical association, rather than causality. Second, some data about the birth weights of children and the maternal gestational weight gain were acquired from the recalling of caregivers, which inevitably resulted in recall bias. To reduce the recall bias, the clinical medical records of child’s vaccination certificate, medical certificate of birth, and maternal health handbook should be taken as the standard, and the subjective recall of the caregivers should be avoided as much as possible. Nonetheless, this large-size epidemiological study involves 5529 children randomly selected from 72 villages across 24 towns in 12 counties of Hunan, and covers the whole preschool age group. Hence, our findings reflect the undernutrition statuses and associated factors of children (age <6 years) in rural Hunan and will help health administrations to lower undernutrition-caused burdens in rural areas.

## Conclusions

The prevalence of stunting, underweight and wasting is low among rural children under age of 6 years in Hunan. Undernutrition of children in this region is affected by birth weight, maternal gestational weight gain, ethnicity of caregivers, education level of caregivers, family size, and family food expenditure. Attention must be focused on strengthening the gestational care and reasonable diet of mothers, and on avoiding nutritional deficiency during pregnancy, which will reduce the occurrence of low birth weight. The local economic development and the education level of caregivers need to be further improved, especially for minorities.

## Data Availability

The datasets generated during and/or analysed during the current study are not publicly available duo to the personal privacy of subjects but are available from the corresponding author on reasonable request.

## Abbreviations

WHO: World Health Organization
HAZ: Length/height for Age Z Score
WAZ: Weight for Age Z Score
WHZ: Weight for Length/height Z Score
BMIZ: Body Mass Index for Age Z Score
AOR: Adjusted Odds Ratio
CI: Confidence Interval
